# A possible manifestation of pancreas divisum–pancreatic pseudocyst in an infant with no apparent history of pancreatitis: a case report

**DOI:** 10.1186/s40792-023-01735-3

**Published:** 2023-09-04

**Authors:** Tokuro Baba, Toru Yamazaki, Masato Sakai, Koichiro Matshuda, Koji Amaya, Mitsuhisa Takatsuki, Yasuhiro Okada

**Affiliations:** 1 Department of Digestive and General Surgery, Graduate School of Medicine, University of the Ryukyus, 207 Uehara, Nishihara, Okinawa 903-0215 Japan; 2 Department of Pediatric Surgery, Toyama Prefectural Central Hospital, 2-2-78 Nishi-Nagae, Toyama, 930-8550 Japan; 3 Department of Internal Medicine, Toyama Prefectural Central Hospital, 2-2-78 Nishi-Nagae, Toyama, 930-8550 Japan; 4 Department of Surgery, Toyama Prefectural Central Hospital, 2-2-78 Nishi-Nagae, Toyama, 930-8550 Japan

**Keywords:** Pancreas divisum, Pancreatitis, Pseudocyst, Endoscopic retrograde cholangiopancreatography (ERCP)

## Abstract

**Background:**

Pancreas divisum (PD), the most common pancreatic anomaly, is caused by the failure of pancreatic bud fusion in the embryo. Although most cases are asymptomatic, it can cause pancreatitis or epigastric pain. We report an unusual case of PD in an infant.

**Case presentation:**

The patient was a 9-month-old girl with no pertinent medical history. She had suffered vomiting and diarrhea for 1 week before transfer to our hospital. Her general condition was poor, and abdominal distention was noted. Blood tests revealed microcytic anemia with normal chemical markers. The parents reported no episode of pancreatitis. Ultrasonography revealed massive ascites, which was later found to be bloody. Enhanced computed tomography and magnetic resonance imaging depicted a cystic lesion, approximately 2 cm in size, anterior to the second portion of the duodenum. During exploratory laparotomy, a pinhole was identified on the cyst wall, which was mistakenly identified as a duodenal perforation, and direct closure was performed. Postoperative levels of serum amylase and inflammation markers were elevated, and the amount of ascites increased, impairing oral feeding. The level of pancreatic enzymes in the ascites was high. Imaging studies were repeated, but the cause of pancreatic fistula was not identified. Conservative therapy, including administration of total parenteral nutrition, antibiotics, and octreotide, was initiated, but the situation did not improve. Three months after admission, endoscopic retrograde cholangiopancreatography showed a thick dorsal pancreatic duct communicating with a hypoplastic ventral duct, which was indicative of PD. Contrast medium leaking from the dorsal duct near the minor ampulla revealed the presence of a pseudocyst. Stenting via the minor papilla was impossible because the minor papilla was obstructed. Instead, a stent was inserted into the ventral pancreatic duct. Endoscopic transgastric drainage of the cyst was effective, and the patient was discharged, 7 months after admission. The patient is healthy, but the gastric stent needs to be replaced regularly.

**Conclusion:**

In children, PD can manifest with pancreatic pseudocyst that causes pancreatic ascites, even in the absence of pancreatitis. This may be a previously unrecognized manifestation of PD in children, and clinicians need to be aware of it.

## Background

Pancreas divisum (PD) is the most common congenital malformation of the pancreas. It results from a failure of fusion of the ductal system of the dorsal and ventral pancreatic buds at 7 weeks of gestation [1]. PD is considered a risk factor for pancreatitis in adults [2]. Pediatric cases of PD have also been reported; however, most reported cases are of relatively older children, and the clinical manifestations in children have been similar to those in adults [3–7]. We report a case of PD with pancreatic pseudocyst formation in an infant with no apparent history of pancreatitis.

## Case presentation

The patient was a 9-month-old girl with no pertinent medical history. She had suffered vomiting and diarrhea for 1 week and was then transferred to our hospital. She looked exhausted and apathetic to her surroundings, and abdominal distention without tenderness was noted. Blood tests revealed microcytic anemia with a hemoglobin level of 5.8 g/dL, but results of biochemical tests, including measurements of hepatic, renal, and pancreatic enzymes, were within normal ranges, and levels of inflammation markers were not elevated (Table 1). Careful documentation of the history with the parents revealed no episode of abdominal pain or blunt trauma to the abdomen since birth.

**Table 1:** Blood test results on admission

Item (unit)	Value	Item (unit)	Value
WBC (/μL)	9900	BUN (mg/dL)	6
Hb (g/dL)	5.8	Cre(mg/dL)	0.15
Plt (/μL)	752,000	AMY(mg/dL)	123
TP (mg/dL)	5.8	Na (mEq/L)	139
Alb (mg/dL)	3.7	K (mEq/L)	4.3
AST (mg/dL)	34	Cl (mEq/L)	107
ALT (mg/dL)	13	Ca (mEq/L)	9.9
T-Bil (mg/dL)	0.6	CRP (mg/dL)	0.11

Blood test on admission showed anemia. Other functions were within the normal range

*WBC* white blood cell, *RBC* red blood cell, *Plt* platelet, *Hb* hemoglobin, *TP* total protein, *Alb* albumin, *AST* aspartate aminotransferase, *ALT* alanine aminotransferase, *T-Bil* total bilirubin, *BUN* blood urea nitrogen, *Cre* creatinine, *AMY* amylase, *Na* sodium, *K* potassium, *Ca* calcium, *CRP* C-reactive protein

**Fig. 1:**
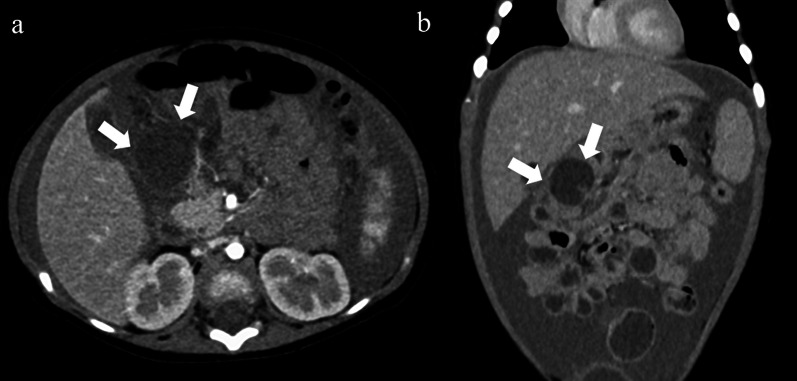
Enhanced CT findings. Enhanced computed tomography showed a cystic lesion (white arrow) anterior to the second portion of duodenum with ascites, as seen in the axial (**a)** and coronal (**b)** views

**Fig. 2:**
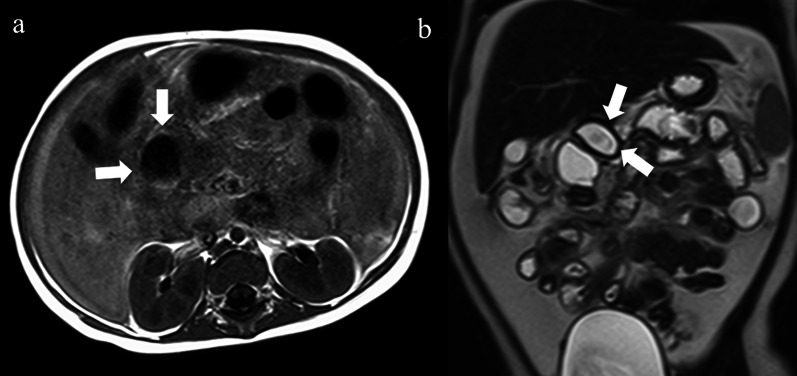
MRI findings. The cyst (white arrows) showed low intensity on T1-weighted magnetic resonance imaging (MRI) (**a**) and high intensity on T2-weighted MRI (**b**)

**Fig. 3:**
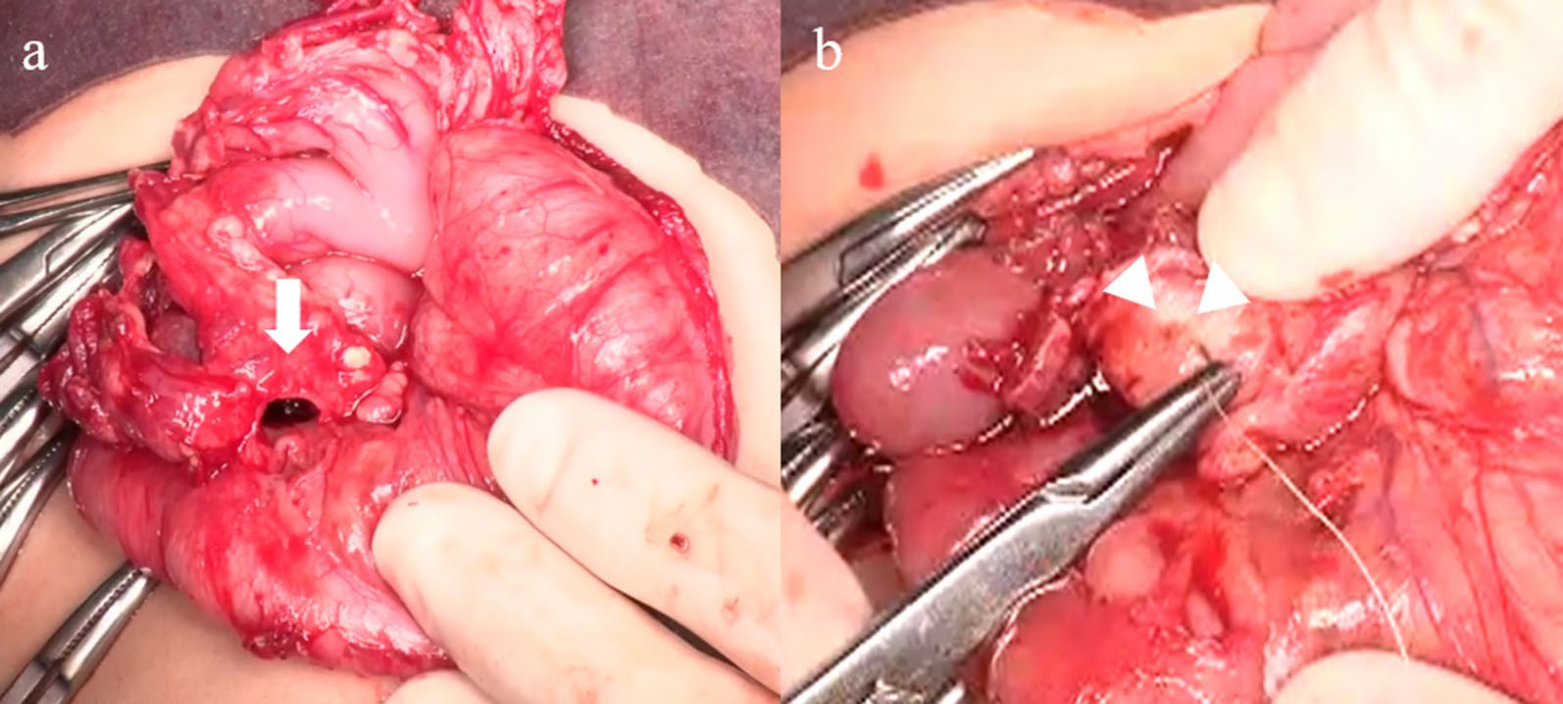
Operative findings. Laparotomy revealed that the omental cavity was filled with clot (white arrow) anterior to the duodenum, along with bloody ascites (**a**). After the cavity was dissected, a pinhole emerged behind the wall, which was mistakenly identified as a duodenal perforation. The hole was closed with absorbable suture and an omental patch (**b**)

Abdominal ultrasonography depicted massive ascites. Enhanced computed tomography (CT) revealed a cystic mass, approximately 2 cm in diameter, anterior to the second portion of duodenum (Fig. 1). The cyst exhibited hypointensity on T1-weighted magnetic resonance imaging (MRI) and high intensity on T2-weighted MRI (Fig. 2). We suspected intra-abdominal ruptured lymphatic malformation at this point. After blood transfusion to correct the anemia, exploratory laparotomy revealed that the peritoneal cavity was filled with bloody ascites and clots, and the omentum was found to cover the second portion of the duodenum. After removing the omentum from the duodenal wall, we found a pinhole in the anterior wall of the cyst, which was misdiagnosed as a duodenal perforation. The pinhole was closed with absorbable sutures and an omental patch (Fig. 3).

Five days after laparotomy, blood tests showed an elevation in serum pancreatic amylase levels (up to 314 U/L). CT depicted a slightly enhanced fluid collection adjacent to the duodenum, which was indicative of abdominal abscess and minor leakage from the closed pinhole. We stopped the patient’s oral feeding, initiated intravenous hyperalimentation, and continued intravenous antibiotics. One week later, the abscess-like lesion appeared smaller on CT, and no leakage was observed in an upper gastrointestinal contrast study.

**Fig. 4:**
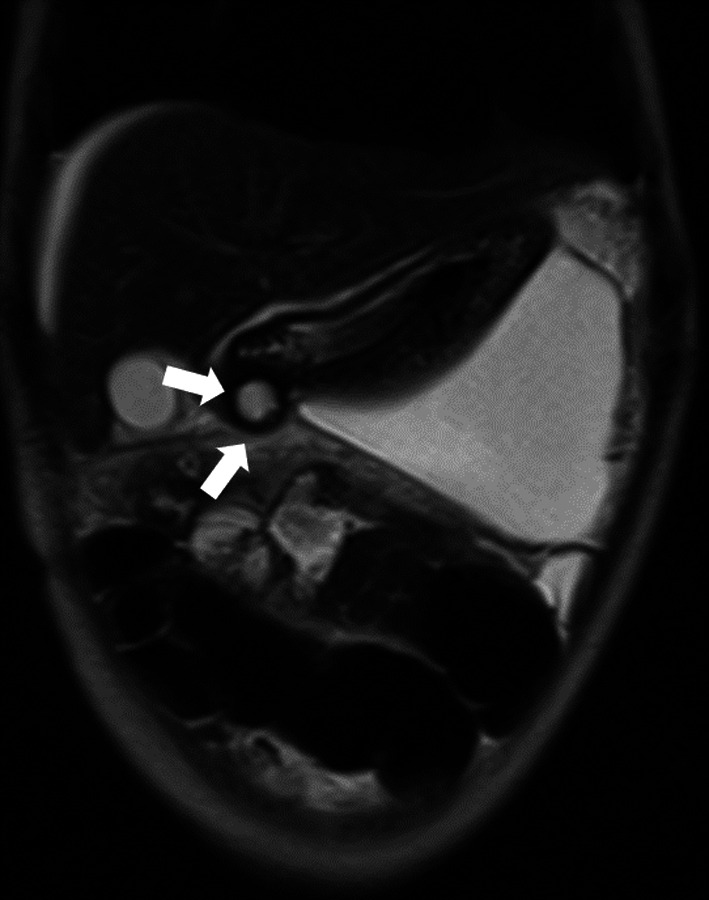
MRI findings. Magnetic resonance imaging showed that the cystic lesion (white arrow) became smaller with increased ascites mainly in the left upper abdomen. The pancreas itself had a normal appearance

We restarted oral feeding; however, the serum amylase level did not normalize. The drainage tube was removed, and then the serum amylase level rose to 1109 U/L. One month after the laparotomy, MRI showed significantly increased ascites in the left upper abdomen, whereas the cystic lesion became much smaller, and the pancreas appeared normal (Fig. 4).

The ascites was drained through a small incision while the patient was under general anesthesia. The amount of pancreatic amylase in the ascites was excessive (11,069 U/L), which suggested that pancreatic fluid had been leaking continuously into the peritoneal cavity. We initiated total parenteral nutrition via a central venous catheter. Treatment with octreotide was also initiated to decrease the secretion of pancreatic fluid. Despite these maximum efforts with conservative therapy, the patient remained unable to tolerate sufficient oral feeding, and total parenteral nutrition was continued.

**Fig. 5:**
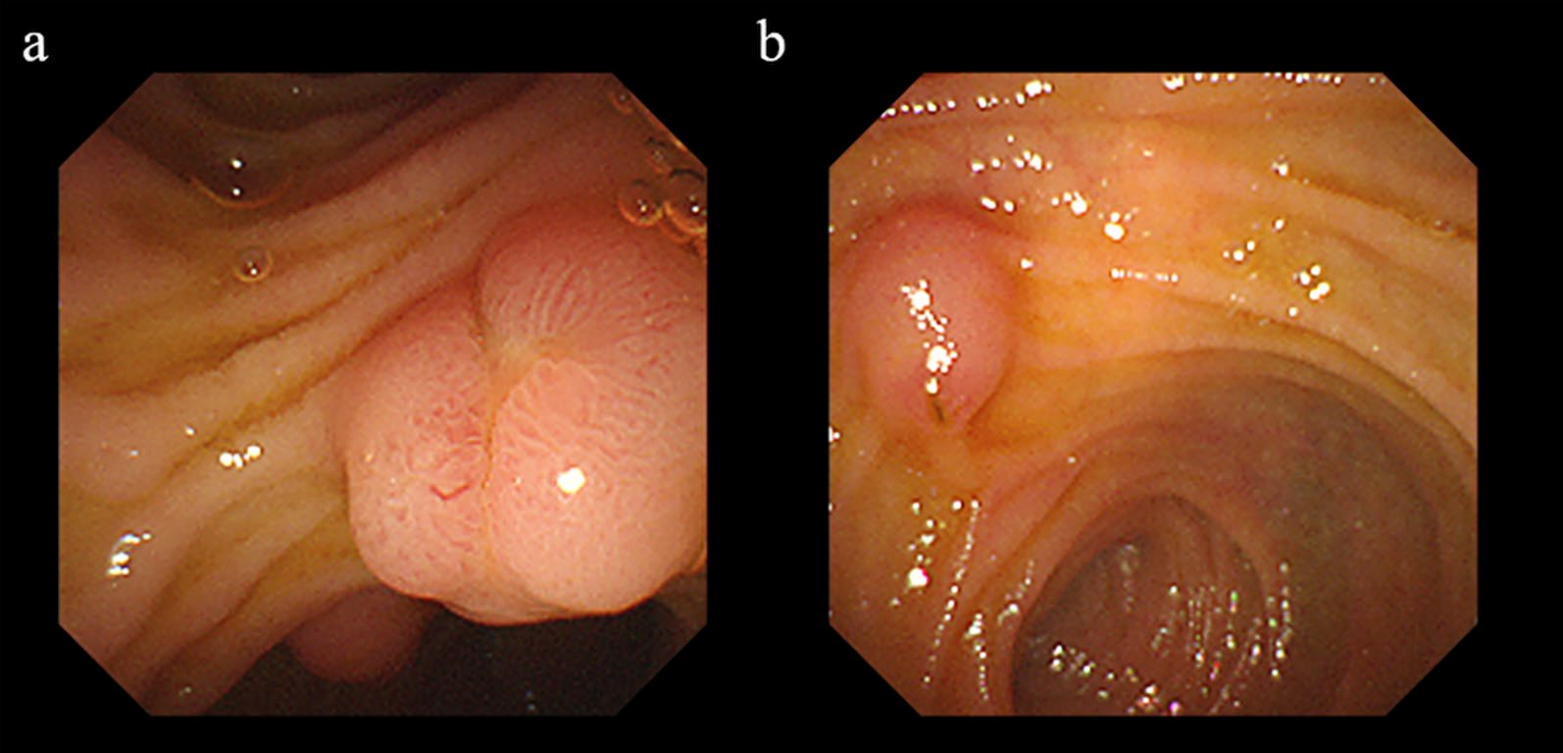
Endoscopic findings of the papilla. The minor papilla (**a**) was more developed than the major papilla (**b**)

**Fig. 6:**
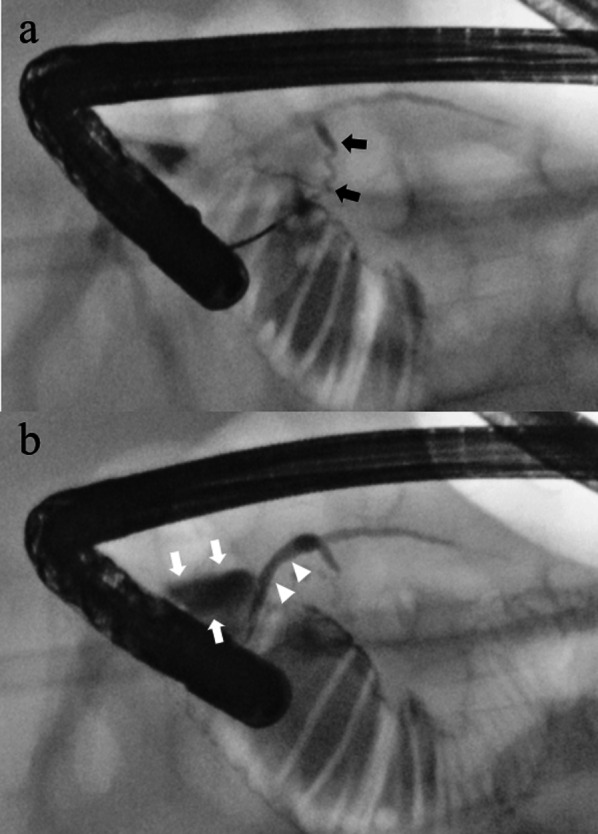
ERCP findings. Endoscopic retrograde cholangiopancreatography revealed a relatively dilated dorsal pancreatic duct (white arrowhead) communicating with a thread-like hypoplastic ventral pancreatic duct (black arrow) (**a**). Contrast media leaked from the dorsal pancreatic duct into a pancreatic pseudocyst (white arrow) near the minor ampulla; the material eventually flowed into the peritoneal cavity (**b**)

Three months after the laparotomy, endoscopic retrograde cholangiopancreatography (ERCP) was performed while the patient was under general anesthesia. Endoscopic findings revealed that the minor papilla was more developed than the major papilla (Fig. 5). Contrast medium injected into the major papilla revealed a thread-like tortuous hypoplastic ventral pancreatic duct in communication with a relatively dilated dorsal pancreatic duct. The dilated dorsal pancreatic duct ended in a pancreatic pseudocyst near the minor papilla. This pseudocyst was misidentified as the anterior wall of the duodenum during laparotomy and an abscess-like lesion on CT after the first surgery. ERCP showed that contrast medium was leaking from the pancreatic pseudocyst into the peritoneal cavity (Fig. 6). These findings were highly suggestive of PD.

Endoscopic placement of the pancreatic stent was planned together with second laparotomy for draining the abdominal pancreatic fluid. We tried to insert the pancreatic stent via the minor papilla, but it was not possible because the minor papilla was almost completely obstructed. Instead, the stent was inserted via the major papilla across the junction of the ventral and dorsal pancreatic ducts, and two drainage tubes were placed in the peritoneum near the surface of the pancreatic pseudocyst.

After the second laparotomy, the serum amylase level normalized, and the patient’s condition improved, although the pancreatic stent spontaneously became dislodged 3 days after its placement. The postoperative level of amylase in the drain fluid gradually increased. The peritoneal drains were replaced 2 weeks after laparotomy, but then the volume of drainage decreased, and the level of serum amylase rose again. Although another pancreatic stent was placed endoscopically through the major papilla, it was not effective in achieving adequate drainage.

**Fig. 7:**
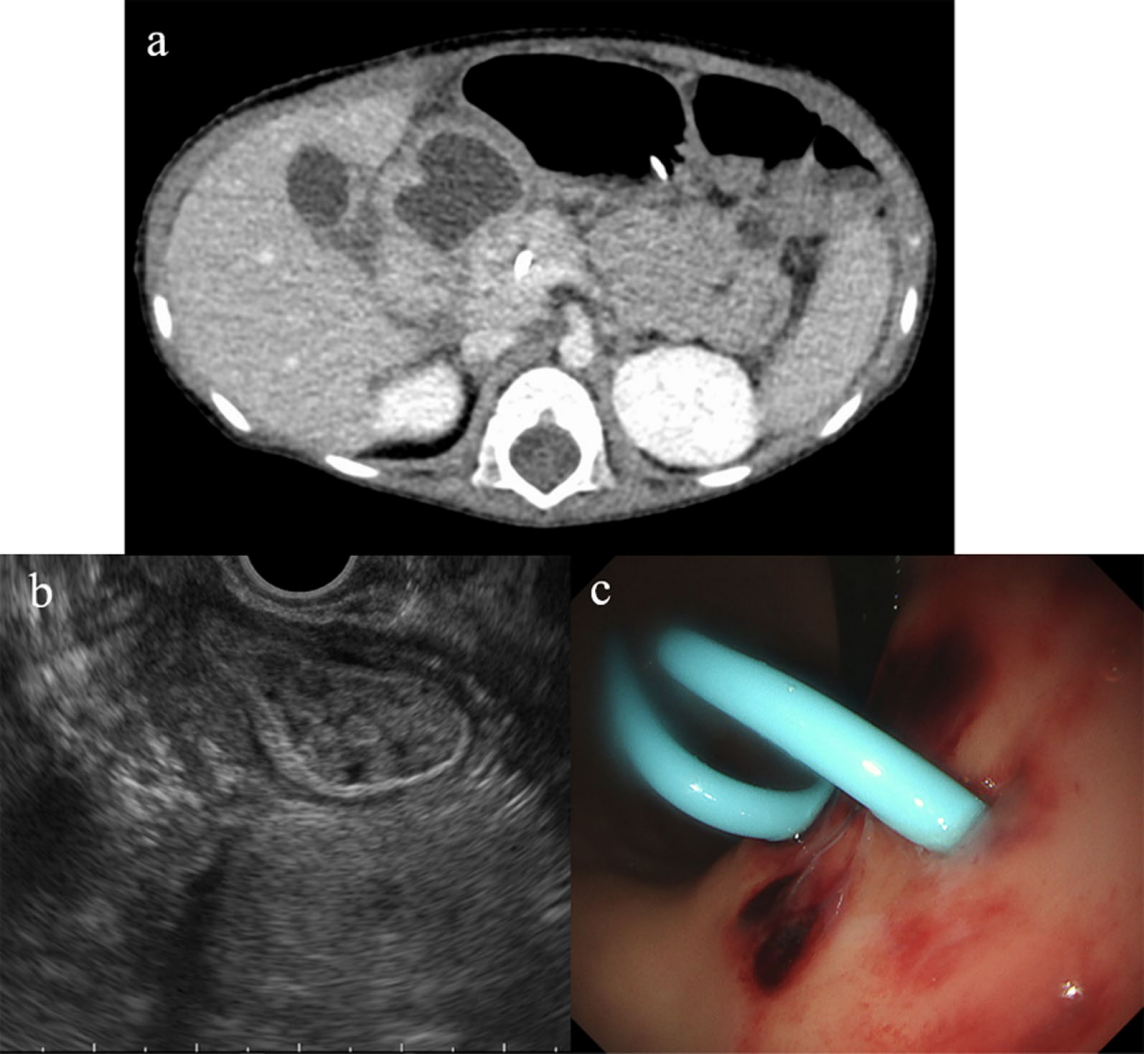
Endoscopic transgastric drainage of the pancreatic pseudocyst. Enhanced computed tomography before endoscopic drainage depicted the pseudocyst directly adjacent to the stomach; it was 28 × 20 mm in size (**a**). It was punctured under endoscopic ultrasound guidance (**b**), and a 7-Fr pigtail catheter was placed in the gastric tract to serve as a stent (**c**)

The anastomosis of pseudocyst with alimentary tract was considered to be ideal, but the wall of the pseudocyst, as depicted by serial ultrasonography, seemed too fragile to perform it. Thus, we selected endoscopic ultrasonography-guided pseudocyst drainage. Enhanced CT had most recently shown that the pseudocyst was 28 × 20 mm in size and adjacent to the stomach. Under the guidance of endoscopic ultrasonography, the pseudocyst was punctured via the gastric wall, and a 7-Fr pigtail catheter was placed to serve as a stent (Fig. 7). The serum amylase level then normalized quickly, and the pancreatic fluid stopped leaking completely. The patient was discharged home; her hospitalization had lasted 7 months.

The patient is currently healthy and putting on weight. However, because the pseudocyst has not shrunk, the pigtail catheter must be exchanged while she is under general anesthesia every 4 months.

## Discussion

PD results when the embryological ventral and dorsal endodermal buds fail to fuse [8, 9]. This anatomical variant was first reported by Opie in 1903 [10]. On the basis of autopsy data, the prevalence was reported to be 6–10% [11, 12]. According to ERCP series, this anomaly is present in 16.4% of adults and 12–20% of children with relapsing pancreatitis [6, 13–16]. With regard to children, no large series have been reported. Neblett et al. studied 10 children with PD; their ages ranged from 2 to 15 years [6]. To our knowledge, our patient is the youngest reported so far.

A classical theory is that in patients with PD, drainage of pancreatic secretions results from the presence of a dominant dorsal duct with a relatively small minor papilla. The consequent increase in intraductal pressure and distention of the dorsal duct can cause abdominal pain and pancreatitis [1]. On the other hand, more than 95% of patients with PD have no symptoms; thus, whether PD causes pancreatitis remains unclear [11, 17, 18].

The annual incidence of pediatric acute pancreatitis was estimated to be approximately 3–13 cases per 100,000 people. Risk factors for acute pancreatitis in infants are more diverse than those in adults, including anatomic change, biliary obstructive, infections, trauma, toxins, metabolic causes, genetic predispositions, and idiopathic etiologies [19]. Unlike adults, children, especially infants, often present with nonfocal abdominal discomfort or irritability [20]. Pancreatic pseudocyst is a well-known complication of acute or chronic pancreatitis, and its overall incidence was reported to be 7% [21]. Pediatric cases of PD in which pancreatic pseudocysts formed after a bout of pancreatitis have been reported [7, 22]. In general, pancreatic pseudocysts form more than 4 weeks after the onset of acute pancreatitis [23]. In our patient, pancreatic pseudocyst was confirmed at initial imaging, but she had had only nonspecific symptoms (diarrhea and vomiting) for only 1 week before admission. She had no signs of pancreatitis, such as a history of abdominal pain, discomfort, or irritability. Although it cannot be denied completely that an episode of pancreatitis might be overlooked by caregivers considering its vague symptom, we believed that the pseudocyst formation resulted directly from PD in this case. We hypothesized that the obstruction of the minor papilla led to blockage of the accessory pancreatic duct and formation of pseudocyst near the minor papilla in the absence of pancreatitis. Pancreatic fluid leaked persistently from the pseudocyst to the peritoneal cavity in this case. Pancreatitis may not have occurred because the activity of pancreatic amylase was low in infants [24, 25]. We have found no report of PD that was characterized by such a clinical course; therefore, this might be a previously unrecognized manifestation of infantile PD.

Magnetic resonance cholangiopancreatography (MRCP) is a noninvasive imaging modality reported 50–70% sensitivity for the diagnosis of PD with [1]. Ueno et al. compared MRCP with ERCP in 97 patients and concluded that MRCP could better demonstrate the normal pancreatic duct and various pancreatic duct abnormalities [26]. PD was detected using MRCP in four of the eight patients (50%) in this series. Unfortunately, MRCP was not performed and an enhanced MRI was conducted several times in our case. If MRCP was performed, the diagnosis could have been established earlier.

At present, ERCP is considered the “gold standard” for the diagnosis of PD [27, 28]. Rosen et al. reported that it could be performed successfully and safely even in children [29]. They analyzed results of ERCP in 184 children, but this population included only three infants younger than 1 year. Although our patient weighed only 7 kg because of growth retardation, ERCP was performed successfully while she was under general anesthesia. The classification criteria for PD reported by Warshaw et al. are well known and considered fundamental [30]. Warshaw et al. reported that the so-called “incomplete PD” is characterized by the presence of a similar filamentous communication between the dorsal and ventral ducts as seen in type 3 PD [30]. ERCP findings in our case revealed a communication between the hypoplastic ventral and thick dorsal ducts. The distal part of the ventral duct near the junction seemed dilated, which is inconsistent with the characteristics of “filamentous communication” reported by Warshaw et al.; however, the dilation was probably secondarily caused due to the increased pressure in the pancreatic duct system. Considering the endoscopic findings of the papilla, it is certain that pancreatic fluid discharge was primarily dependent on the ventral duct. Thus, the case was consistent with PD.

We believe that ERCP is essential for the diagnosis of PD even in infants, despite certain obstacles such as the need of general anesthesia and small devices for infants. If pancreatic ascites of unknown origin is depicted by other imaging modalities, ERCP should be considered.

Obstruction of the minor papilla appears to be the central pathogenesis of symptomatic PD [31]. The main goals of treatment are to improve the flow of pancreatic fluid and to reduce intraductal pressures. The endoscopic approach is a less invasive and standard approach. ERCP, including papillotomy of the minor papilla, with or without stent implantation, is the first choice of therapeutic intervention, even in children [32–34]. According to the study by Pan et al., 114 ERCPs were performed in 46 children with PD. Among the procedures, 40 (35.1%) were endoscopic papillotomy; 95 (91.3%), pancreatic duct stenting; 44 (63.0%), pancreatic duct dilation; and 68 (73.9%), stone extraction [34]. The post-ERCP clinical remission rate was 87%. In our patient stent insertion via the minor papilla seemed reasonable for preventing the leakage of pancreatic fluid into the peritoneal cavity; however, stent insertion into the minor papilla was impossible, and it was inserted through the major papilla instead but did not improve her condition.

As operative management in adults with pancreatic pseudocyst, the pseudocyst can be anastomosed to the alimentary tract if the cyst wall is rigid and not inflamed [35]. However, in our patient, the wall of the pseudocyst as depicted by serial ultrasonography which seemed too fragile to tolerate anastomosis. We ultimately performed internal drainage through the endoscopic approach. Endoscopic ultrasonography-guided pseudocyst drainage through the gastric wall was successful; the patient’s condition improved, and she was finally discharged home. At present, however, the stent must be replaced regularly because the pseudocyst has not shrunk. Owing to the direct communication between the cyst and dorsal pancreatic duct, which is crucial for the passage of almost all pancreatic fluid, the used 7-Fr pigtail catheter might have been relatively small considering the amount of pancreatic fluid flowing into the cyst. When the patient reaches adulthood, the pancreatic stent might be inserted via the minor papilla. Additionally, lumen apposing metal stents (LAMSs), which are often used in patients with pancreatic cyst, can be a treatment of choice in the future [36]. Other radical surgery, including pancreatoduodenectomy with or without pylorus preservation or duodenum-preserving resection of the pancreatic head, has also been reported [32]. However, these surgical procedures have been performed only in limited numbers of adults with PD. We consider these procedures too invasive for younger children.

We encountered an infant with suspected PD. Although she presented with nonspecific symptoms, persistent leakage of pancreatic fluid from the pseudocyst was observed. After recording the medical history of the patient, it was likely that pancreatitis was not associated with pancreatic pseudocyst formation. Owing to the unusual presentation of PD in this case, it took us a long time to make the diagnosis due to the delay in performing ERCP. Although the therapeutic strategies are currently limited because of the small body size, the development of alternative strategies in the future is warranted. Only few studies have focused on PD in infants, and further studies are necessary to determine its pathophysiology and treatment.

## Conclusions

We diagnosed PD with pancreatic pseudocyst in an infant without any apparent history of pancreatitis. Pancreatic fluid leaked persistently into the peritoneal cavity. Endoscopic ultrasonography-guided pseudocyst drainage through the gastric wall was effective in resolving this leakage. In children with PD, especially infants, pancreatic pseudocysts can form as a result of occlusion of a pancreatic duct without pancreatitis. This might be a previously unrecognized manifestation of PD, and clinicians need to be aware of it.

## Data Availability

Not applicable.

## Abbreviations

CT: Computed tomography
ERCP: Endoscopic retrograde cholangiopancreatography
PD: Pancreas divisum
MRI: Magnetic resonance imaging
MRCP: Magnetic resonance cholangiopancreatography
